# Influence of Radiofrequency Electromagnetic Fields on the Fertility System: Protocol for a Systematic Review and Meta-Analysis

**DOI:** 10.2196/resprot.9102

**Published:** 2018-02-08

**Authors:** Nasibeh Roozbeh, Fatemeh Abdi, Azadeh Amraee, Zahra Atarodi Kashani, Leili Darvish

**Affiliations:** ^1^ Mother and Child Welfare Research Center Hormozgan University of Medical Sciences Bandar Abbas Islamic Republic Of Iran; ^2^ Students Research Committee Nursing and Midwifery Faculty Shahid Beheshti University of Medical Sciences Tehran Islamic Republic Of Iran; ^3^ Department of Medical Physics School of Medicine Iran University of Medical Sciences Tehran Islamic Republic Of Iran; ^4^ Iranshahr university of medical sciences Iranshahr Islamic Republic Of Iran; ^5^ Zabol Medicinal Plants Research Center Zabol University of Medical Sciences Zabol Islamic Republic Of Iran; ^6^ Research & Technology Department Fertility and Infertility Research Center Hormozgan University of Medical Sciences Bandar Abbas Islamic Republic Of Iran

**Keywords:** electromagnetic field, fertility, reproduction

## Abstract

**Background:**

Due to the increased number of users of mobile phones, tablets, and other devices over the past few years, concerns about the potential impact of mobile phones on health are growing. The influence of mobile phone exposure on male fertility has been studied in recent years. Other research has shown that electromagnetic fields (EMFs) increase macrophages in the corpus luteum and growing follicles. Due to conflicting results among studies and since no systematic review has been performed to analyze the effects of radiofrequency EMF exposure from electronic devices on the fertility system in recent years, this evidence-based study is necessary.

**Objective:**

The main objectives of this study are to determine the best evidence associated with the influence of radiofrequency EMFs on the fertility system and to provide insight into a potential mechanism using our observations.

**Methods:**

In this systematic review, the databases and gray literature will be searched with no language and date limitation. The following databases will be searched: Cochrane Library, MEDLINE, PubMed, EMBASE, CINAHL, ProQuest, Scopus, Science Direct, Google Scholar, and other Persian databases. The combination of the Medical Subject Heading terms “radiofrequency electromagnetic” and “male reproductive system” or “female reproductive system” will be searched. Observational study designs will be included but case reports, case series, reviews, and letters to the editor will be excluded. Papers selected for retrieval will be evaluated by two independent referees for methodological validation before entering a review using the Newcastle-Ottawa Scale for nonrandomized studies and cohort studies.

**Results:**

The results of this study will be submitted to a peer-reviewed journal for publication and also presented at PROSPERO.

**Conclusions:**

This systematic review will provide evidence-based data on the effect of radiofrequency EMFs on the fertility system. This article will also classify the harmful effect of radiofrequency waves on primary and secondary infertility. This study could be useful for decreasing infertility. This is important because the rate of infertility is growing, leading to negative outcomes for couples and the health care system.

**Trial Registration:**

PROSPERO CRD42017072462; https://www.crd.york.ac.uk/prospero/display_record.php?RecordID=72462 (Archived by WebCite at http://www.webcitation.org/6wjiE9R2q)

## Introduction

Concerns about the potential impact of mobile phones on health are growing due to the increased use of the mobile phones, tablets, and other devices over the past few years. Mobile phones, wireless telephones, mobile base stations, and power lines are some of the main sources of our daily exposure to radiofrequency electromagnetic fields (RF-EMFs). There are more than 2 billion mobile phones in use worldwide [1-3].

Exposure to RF energy depends on the frequency of the mobile phone used. The most common frequency of phones used in the United States is 900 to 1900 MHz, while in many parts of the world, phones work at frequencies from 850 to 1800 MHz. The higher the frequency, the higher the energy. Radiant energy is absorbed by 3 main mechanisms in the human body: (1) Aural effect; the body receives and absorbs the RF signal depending on the size of the body part and the signal wavelength; (2) RF signal binds with tissue; and (3) absorption intensification [4,5].

The effects of electromagnetic radiation (EMR) can be divided into two main categories: thermal properties and nonthermal properties. Thermal properties are caused by the increase in temperature due to the energy absorption of oscillating electric fields. This can lead to heat in the exposed parts of the body. Thermal effects are calculated in terms of specific absorption rate. The specific absorption rate depends largely on the antenna, location, and frequency settings [6]. Some studies show that human exposure to RF waves can cause cognitive and behavioral impairments and decreased learning and memory. Significant thermal effects may be associated with adverse health effects such as problems with sleep, hearing, reproduction, impairment of the nervous system and increased cancer risk [7].

Infertility is defined as the inability to get pregnant after one year of intercourse without the use of contraceptive methods, and it affects 15% of couples worldwide. Male infertility is the cause of 30-50% of infertility, of which 30-40% of the causes are referred to as sperm disorders [8]. The influence of mobile phone exposure on male fertility has been studied in recent years [9]. In normal physiological conditions, spermatogenesis is a balanced process of maturation, cell division, and storage, which is particularly susceptible to environmental stimuli and chemicals. The mechanism is unclear, but its hypothesized that the component involved is the cytoskeleton which consists of charged proteins. The cytoskeleton is a structural and functional part of the cell that plays a main role in motility of the sperm and actively participates in the morphological alterations that happen during mammalian spermatogenesis [9,10].

A study of the effect of EMFs on female rats using transmission electron microscopy showed the increased existence of numerous drops of lipid in patches and luteal cells, as well as an increase in the number of autophilic antibodies and macrophages in some granulosa cells [11]. Other researchers showed that EMFs increase macrophages in the corpus luteum and growing follicles, and they believe that EMFs increase apoptosis in mice ovaries. In addition, most researchers showed that EMFs damage stromal cells in the uterus and uterine tubes through apoptosis of the glandular epithelium, ovarian cortex, and luminal epithelium [12,13].

It is necessary for future research to explore the safety criteria of RF-EMR. As electronic devices that emit RF-EMR are used very close to the body, RF shielding could be used in electronic devices to block RF-EMF waves and increase distance from them. Systematic review papers are a type of review that analyze the findings of other studies and provide the best evidence for a decision about a health approach [14]. Some studies demonstrated the potential harmful effects of RF on various sperm parameters, such as sperm motility, due to mobile phone usage [15]. Due to conflicting results among published research and since no systematic review has been performed to analyze the effects of RF-EMF exposure from other electronic devices on the fertility system in recent years, this evidence-based study is necessary.

The aims of this study are to clarify the best evidence associated with the influence of RF-EMFs on the fertility system and to make observations that could provide insight on a potential mechanism. The other objective of this study is to determine the effect of an RF-EMF on primary or secondary infertility.

## Methods

### Inclusion Criteria

This systematic review will include studies with infertile couples, defined by the inability to get pregnant after one year of intercourse without the use of contraceptive methods, of which the male and female are 18-65 years of age and use a device omitting RF-EMF for various exposures. We will include studies that used various methods of analysis to assess the reproductive system, including hormonal assessments, diagnostic imaging techniques, biopsies, and spermatograms.

This systematic review will only include human studies and the intervention can be the use of any device that exposed a user to RF-EMFs for any frequency and duration. There will be no restrictions on exposure condition, type of signal device, distance, exposure time, and location of participant.

All studies with or without the control group will be included in this study.

Any observational study designs will be included. Case reports, case series, reviews, and letters to the editor will be excluded. Articles with incomplete data will be excluded from the study. We will try to contact the authors of studies that are related but not accessible by email.

### Outcomes

This systematic review will consider papers that include a rate of change in reproductive system parameters (sperm, endocrine parameters [luteinizing hormone, follicle-stimulating hormone, and testosterone], testis, and ovary function) after the use of devices with an RF-EMF as primary outcomes. The potential mechanism of RF-EMF on the fertility system and primary or secondary infertility will be the secondary outcome of this systematic review.

### Search strategy

In this systematic review, databases and gray literature will be searched without language or date restrictions. The following databases will be searched: Cochrane Library (Wiley), MEDLINE (Ovid), PubMed, EMBASE, CINAHL (EBSCO), ProQuest, Scopus, Science Direct, Google Scholar, and other Persian databases. All databases will be scanned for articles from the year 2000 until the present.  The reference lists of articles and reports will be checked. The gray literatures includes the International Clinical Trials Registry Platform and ProQuest Dissertations & Theses Global. The combination of the Medical Subject Headings terms “radiofrequency electromagnetic” and “male reproductive system” or “female reproductive system” will be used for our search. To ensure the inclusion of all the related articles, the search will be sensitive and accurate. The title and abstract of the articles will be evaluated and any disagreement over the inclusion of an article in the review will be resolved through discussion between the authors. The full-text of articles will be assessed to meet the objectives of this systematic review.

### Quality assessment

Papers selected for retrieval will be evaluated by two independent referees for methodological validation before entering the review using the Newcastle-Ottawa Scale to evaluate the quality of cohort studies and nonrandomized controlled trials [16]. Any disagreements that arise between reviewers will be resolved through discussion or with a third reviewer.

### Data extraction

The data from the articles used in this study will be extracted by two independent reviewers using the standard data extraction tool, Joanna Briggs Institute System for the Unified Management, Assessment and Review of Information. The data extracted will comprise of specific details about the populations, interventions, study methods, and results which are important to the objectives of the review. Any disagreements that arise will be resolved through discussion between the reviewers or with a third reviewer. Authors of papers will be contacted to request missing or additional data where required. We will try to contact the corresponding authors of studies by email if it is necessary to obtain data missing from published articles.

### Data synthesis

Analyses will be conducted using the STATA V.12 software. Heterogeneity will be evaluated with the I^2^ statistic and chi-square (χ^2^) test (recommended by the Cochrane Handbook for Systematic Reviews of Interventions). We will explain the I^2^ statistic using the following example. When substantial heterogeneity (I^2^>50%) is evident among the articles, the results of this study will be presented in the text qualitatively. The author’s decision to use the random-effects model will be based on an understanding of whether all included trials share a common effect size, not only for results of tests but also for statistical heterogeneity. For classified data, the effect sizes are calculated as odds ratios, and for continuous data, the weighted average difference and their 95% CI are analyzed. By using the standard chi-squared, non-correlation will be evaluated. The results of this article will be presented in the appropriate tables and figures. Publication bias will be explored using a funnel plot and Begg's and Egger's tests. If it is possible, this systematic review will be performed using subgroup meta-analysis based on the type of device and exposure time disagreement.

## Results

This is a protocol for a systematic review, so the results are not presented. The results of this study will be submitted to a peer-reviewed journal for publication and also presented at PROSPERO (international prospective register of systematic reviews). The PROSPERO registration number is CRD42017072462.

## Discussion

The purpose of systematic reviews is to present a comprehensive summary of articles related to a study question and to provide solutions to disagreeing results between studies. While the greatest source of evidence for informed decisions is provided by systematic reviews and meta-analyses, to the best of our knowledge, this will be the first systematic review evaluating the radiofrequency electromagnetic field on fertility. This systematic review will provide evidence-based data on the effect of radiofrequency electromagnetic waves on fertility system. This article will also classify the harmful effect of RF waves on primary and secondary infertility and will validate the use of radiofrequency electromagnetic devices during reproductive period. This study could be useful for decreasing infertility. This is important due to the growing rate of infertility, which has negative outcomes for couples and health care system. The absence of studies evaluating the effects of radiofrequency electromagnetic waves on the fertility system will be a limitation of this study.

## Abbreviations

EMF: electromagnetic field
EMR: electromagnetic radiation
RF: radio frequency

## References

[1] Poulletier DGF, Haro E, Hurtier A, Taxile M, Athane A, Ait-Aissa S, Masuda H, Percherncier Y, Ruffié G, Billaudel B, Dufour P, Veyret B, Lagroye I (2012). Effect of in utero wi-fi exposure on the pre- and postnatal development of rats. Birth Defects Res B Dev Reprod Toxicol.

[2] Khullar S (2012). Impact of Electromagnetic Waves Generated by Cellular Phones on Male Fertility: A Review. Asian Journal of Biomed and Pharma.

[3] Sivani S, Sudarsanam D (2012). Impacts of radio-frequency electromagnetic field (RF-EMF) from cell phone towers and wireless devices on biosystem and ecosystem—a review. Biology and Medicine.

[4] D'Andrea JA, Emmerson RY, Bailey CM, Olsen RG, Gandhi OP (1985). Microwave radiation absorption in the rat: frequency-dependent SAR distribution in body and tail. Bioelectromagnetics.

[5] Deepinder F, Makker K, Agarwal A (2007). Cell phones and male infertility: dissecting the relationship. Reprod Biomed Online.

[6] Koops FB (1998). New ICNIRP guidelines for human exposure to 50 Hz E- and M-fields. Health Phys.

[7] Davis D (2015). Environmental Health Trust.

[8] Godmann M, Lambrot R, Kimmins S (2009). The dynamic epigenetic program in male germ cells: Its role in spermatogenesis, testis cancer, and its response to the environment. Microsc Res Tech.

[9] Agarwal A, Deepinder F, Sharma RK, Ranga G, Li J (2008). Effect of cell phone usage on semen analysis in men attending infertility clinic: an observational study. Fertil Steril.

[10] Agarwal A, Desai NR, Ruffoli R, Carpi A (2008). Lifestyle and testicular dysfunction: a brief update. Biomed Pharmacother.

[11] Susa M, Pavicić I (2007). [Effects of radiofrequency electromagnetic fields on mammalian spermatogenesis]. Arh Hig Rada Toksikol.

[12] Roshangar L, Soleimani RJ (2004). Electron microscopic study of folliculogenesis after electromagnetic field exposure. Journal of Reproduction and Infertility.

[13] Soleimani Rad J, Rowshangar L, Karimi K (2001). The effect of Electromagnetic field on Fallopian Tube.

[14] Asghari A, Khaki AA, Rajabzadeh A, Khaki A (2016). A review on Electromagnetic fields (EMFs) and the reproductive system. Electron Physician.

[15] Yildirim M, Kaynar M, Badem H, Cavis M, Karatas O, Cimentepe E (2015). What is harmful for male fertility: cell phone or the wireless internet?. The Kaohsiung journal of medical sciences.

[16] Stang A (2010). Critical evaluation of the Newcastle-Ottawa scale for the assessment of the quality of nonrandomized studies in meta-analyses. Eur J Epidemiol.

